# Epidemiological approaches to frailty - contributions from the ESTHER study

**DOI:** 10.1186/1753-6561-7-S4-S9

**Published:** 2013-08-23

**Authors:** Hermann Brenner, Kai-Uwe Saum

**Affiliations:** 1 Division of Clinical Epidemiology and Aging Research, German Cancer Research Center, Heidelberg, Germany

## 

Frailty is an emerging concept in gerontology. Two popular approaches to quantify frailty include the deficit accumulation approach which quantifies frailty by the proportion of deficits from a list of symptoms, signs, diseases and functional limitations present (the so-called “frailty index” [FI]), and a rules-based approach which defines frailty by the presence of at least 3 of 5 criteria (exhaustion, unintentional weight loss, low grip strength, low gait speed, low physical activity) (Fried frailty criteria). We employed both concepts in the context of ESTHER, a cohort study of older adults in Saarland, Germany.

Baseline data from ESTHER (N: 9,886; age: 50-75) were used to create a FI through a deficit accumulation approach. Baseline FI was evaluated by age and sex and related to 10-year survival. Below 60 years of age, men had a higher FI than women. However, mean FI showed a stronger increase with age among women (3.1% per year) than among men (1.7% per year) and was higher among women at older age. Relative mortality (95% confidence interval) was 1.08 (0.84-1.39), 1.32 (1.05-1.66), 1.77 (1.41-2.22), and 2.60 (2.11-3.20) for the 2^nd^, 3^rd^, 4^th^ and 5^th^ compared to the 1^st^ quintile of the FI. There was a strong dose-response relationship between the FI and mortality among both men and women and both younger (<65 years) and older subjects.

Frailty was operationalized using modified Fried frailty criteria among 3,112 participants aged 58 years or older who underwent extensive geriatric assessment during 8-year follow-up of the study. Frailty criteria were categorised either according to quintiles or using population-independent cut-points, which were derived from the literature. Although frailty prevalence was higher with the latter (8.9%) than with the former (6.5%) approach, agreement and correlation of frailty classification by both approaches was high (Kappa = 0.75, r_Spearman_ = 0.84). Sex differences in frailty prevalence were more pronounced using the population-independent approach. Similarly strong associations with socio-demographic, lifestyle and medical factors such as older age, female sex, smoking, and obesity were seen for both approaches.

Both the deficit accumulation approach and the rules-based approach are feasible and highly informative in epidemiological ageing research. The former is applicable and highly predictive of mortality even below 65 years of age. Modification of the Fried index by using population independent cut-points of frailty criteria may enhance comparability across studies.

